# Growth of *Chlamydia pneumoniae* Is Enhanced in Cells with Impaired Mitochondrial Function

**DOI:** 10.3389/fcimb.2017.00499

**Published:** 2017-12-05

**Authors:** Nadja Käding, Inga Kaufhold, Constanze Müller, Marta Szaszák, Kensuke Shima, Thomas Weinmaier, Rodrigo Lomas, Ana Conesa, Philippe Schmitt-Kopplin, Thomas Rattei, Jan Rupp

**Affiliations:** ^1^Department of Infectious Diseases and Microbiology, University of Lübeck, Lübeck, Germany; ^2^Research Unit Analytical BioGeoChemistry, Helmholtz Center Munich, Neuherberg, Germany; ^3^Division of Computational Systems Biology, Department of Microbiology and Ecosystem Science, University of Vienna, Vienna, Austria; ^4^Genomics of Gene Expression Lab, Centro de Investigaciones Príncipe Felipe, Valencia, Spain; ^5^Microbiology and Cell Science, IFAS, University of Florida, Gainesville, FL, United States

**Keywords:** mitochondria, hypoxia, *Chlamydia pneumoniae*, metabolism, host-pathogen interaction

## Abstract

Effective growth and replication of obligate intracellular pathogens depend on host cell metabolism. How this is connected to host cell mitochondrial function has not been studied so far. Recent studies suggest that growth of intracellular bacteria such as *Chlamydia pneumoniae* is enhanced in a low oxygen environment, arguing for a particular mechanistic role of the mitochondrial respiration in controlling intracellular progeny. Metabolic changes in *C. pneumoniae* infected epithelial cells were analyzed under normoxic (O_2_ ≈ 20%) and hypoxic conditions (O_2_ < 3%). We observed that infection of epithelial cells with *C. pneumoniae* under normoxia impaired mitochondrial function characterized by an enhanced mitochondrial membrane potential and ROS generation. Knockdown and mutation of the host cell ATP synthase resulted in an increased chlamydial replication already under normoxic conditions. As expected, mitochondrial hyperpolarization was observed in non-infected control cells cultured under hypoxic conditions, which was beneficial for *C. pneumoniae* growth. Taken together, functional and genetically encoded mitochondrial dysfunction strongly promotes intracellular growth of *C. pneumoniae*.

## Introduction

Mitochondria play a major role in the generation of energy but also regulate cell death, production of reactive oxygen species (ROS), and sensing of glucose and oxygen (Heikal, 2010). Thus, mitochondrial function is a prerequisite for cell homeostasis and mitochondrial dysfunction has been linked to various pathologies e.g., cancer cell formation that is characterized by inhibition of cytochrome c induced apoptosis (Vaughn and Deshmukh, 2008). Moreover, mitochondrial dysfunction is a consequence of low oxygen concentrations referred to as hypoxia arising at pathophysiological sites. Thereby, cells need to metabolically adapt to hypoxia by switching the metabolism from oxidative phosphorylation (OXPHOS) to anaerobic glycolysis to maintain ATP supply. Mitochondrial dysfunction can be defined as abnormality in generation of ATP by OXPHOS, ROS production, regulation of apoptosis, regulation of cytoplasmic and mitochondrial matrix calcium, synthesis and catabolism of metabolites as well as mitochondrial trafficking (Brand and Nicholls, 2011).

A decrease in maximal respiration indicates a mitochondrial dysfunction (Brand and Nicholls, 2011). Impairment of the respiratory chain leads to an increased glycolysis, supporting tumor cell growth, and proliferation (Warburg, 1956). Further, an increased mitochondrial membrane potential, termed mitochondrial hyperpolarization, prevents apoptosis (Michelakis, 2008). Mitochondria mediate innate immune responses at different levels by supporting cellular metabolic reprogramming and the cytosolic immune signaling cascades (Monlun et al., 2016). Activation of macrophages and dendritic cells by pro-inflammatory stimuli causes a metabolic switch away from OXPHOS toward glycolysis (Kelly and O'Neill, 2015).

Numerous studies have described apoptosis regulation through the mitochondrial pathway in *Chlamydia* (Sharma and Rudel, 2009), but still there is a lack of information regarding other mitochondrial functions involved in chlamydial infection. It has previously been shown that *Chlamydia trachomatis* induces ROS production, which is beneficial for their development (Abdul-Sater et al., 2010; Boncompain et al., 2010; Chumduri et al., 2013). Further, *C. trachomatis* infection preserved the mitochondrial network (Chowdhury et al., 2017). Besides the production of ROS in *Chlamydia pneumoniae*, mitochondrial dysfunction is implicated with inflammasome activation in *C. pneumoniae* infected macrophages (Shimada et al., 2011).

Although the obligate intracellular bacterium *C. pneumoniae* relies on host cell metabolism, little is known about the influence of mitochondrial respiration on intracellular growth and progeny of *C. pneumoniae*.

While chlamydiae have a limited metabolic capacity due to their small genome size, they are still able to perform certain metabolic steps through the interconversion of metabolites obtained from host cells (Stephens et al., 1998; Kalman et al., 1999). Whole genome sequencing revealed metabolic genes of glycolysis and pentose phosphate pathway (PPP) (Stephens et al., 1998; Iliffe-Lee and McClarty, 1999; Kalman et al., 1999; McClarty, 1999). Furthermore, the chlamydial energy generation is maintained by functional components of the electron transport chain (ETC) and their own V-ATP synthase (McClarty, 1999; Gerard et al., 2002; Skipp et al., 2005). Finally, the chlamydial genome contains a cytochrome bd oxidase, which has been associated with microaerobic respiration in *Coxiella burnettii* under low oxygen conditions (Omsland et al., 2009).

In our model we focused on infections with *C. pneumoniae*, which showed a significantly enhanced growth under hypoxic conditions (Juul et al., 2007; Rupp et al., 2007; Szaszak et al., 2013). In this study we aimed to assess the mitochondrial activity during intracellular *C. pneumoniae* infection and investigated how mitochondrial dysfunction interferes with chlamydial growth and progeny. We utilized hypoxia as a model for analyzing mitochondrial dysfunction in a physiological condition and further targeted the F_0_-subunit of the host cell ATP synthase to validate the findings in a more defined setting.

## Materials and methods

### Cell culture and infection of *C. pneumoniae*

2.5 × 10^5^ HEp-2 cells (ATCC CCL-23) per 6-well were cultured in RPMI 1640 (Invitrogen) medium supplemented with 5% fetal bovine serum (FBS) (PAN-Biotech), 2 mM L-glutamine (Lonza) and 1x non-essential amino acids (HyClone). HEp-2 cells were infected with 0.5 inclusion forming units (IFU) *C. pneumoniae* strain CWL029 (ATCC VR-1310) per cell by centrifugation with addition of 0.1 μg/mL cycloheximide (Sigma-Aldrich), this was also applied to uninfected cells. Cell numbers were identical between infected and non-infected cells. After infection, the cells were cultivated for the indicated time points at 20% oxygen (normoxia) or 2% oxygen (hypoxia) in a hypoxia chamber (Toepffer Lab Systems) equipped with an oxygen and carbon dioxide sensor.

### Chlamydial recovery

To determine the burden of infectious *C. pneumoniae*, titration experiments were performed. Infected HEp-2 cells were detached with a cell scraper and resuspended in fresh growth medium. Serial dilutions of suspension were inoculated in confluent cycloheximide-treated (1 μg/mL) HEp-2 cell monolayers with the assistance of centrifugation. After 48 h, development of chlamydial inclusions was analyzed on methanol-fixed slides using FITC-labeled monoclonal chlamydial-LPS antibodies (Dako). Chlamydial recovery was calculated as IFU/mL by observation of 10 microscopy fields (40 × magnification) with a fluorescence microscope (Axiovert 25, Zeiss) using a LD Achroplan 40x/0.60 Korr objective (Zeiss).

### Immunofluorescence staining of *C. pneumoniae*

Direct immunofluorescence staining was used to determine the infection rate as well as to analyze cell morphology and chlamydial inclusions. Cells grown on coverslips were transferred to a 24-well plate and fixed with methanol at −20°C. Afterwards the methanol was removed and coverslips were dried at 37°C. Ten microliters of FITC-conjugated Chlamydia specific antibody (IMAGEN Chlamydia Kit, Oxoid) was applied and cells were incubated at 37°C. After washing, coverslips were fixed on microscope slides with Mounting Fluid (Oxoid). Specimens were visualized with a fluorescence microscope (BZ-9000, Keyence) equipped with a S Plan Fluor 40×/0.60 objective (Nikon). Chlamydial inclusions appeared green and the cellular compartments red.

### Metabolic analysis of *C. pneumoniae*-infected HEp-2 cells

To obtain a metabolic profile of *C. pneumoniae*-infected HEp-2 cells under normoxic and hypoxic conditions, we performed non-targeted metabolomic analysis applying ultra-high resolution mass spectrometry. Metabolites were purified and analyzed as described previously (Muller et al., 2013). Ten independent biological replicates were prepared, carried out on three different days. Unsupervised multivariate statistics (Principal Component Analysis, PCA) were applied for visualization and exploration of naturally occurring clusters. Discriminative metabolites were extracted by partial least square discriminative analysis (PLS-DA; Simca-P 9, Umetrics, Umea, Sweden) and approved by non-parametric Wilcoxon-Mann-Whitney test. Masses with a variable importance in projection (VIP) >1 and *p* < 0.5 were considered as significant and were further analyzed by KEGG pathway.

### Transcriptional analysis of *C. pneumoniae*

To determine the transcriptome of *C. pneumoniae* under normoxic and hypoxic conditions, total RNA was isolated by NucleoSpin RNA II kit (Macherey Nagel). Human rRNA was depleted by RiboZero rRNA removal kit (Epicentre) in order to enrich bacterial RNA. Human rRNA depleted RNA was send to Vertis Biotechnologie AG (Freising/Germany) for cDNA synthesis (250–400 bp). The tagged cDNA libraries were pooled and single-read sequencing (read length 50 bp) was performed on Illumina HiSeq 2000 by BGI-Hong Kong.

Illumina reads were mapped to the *C. pneumoniae* genome (AE001363.1; Benson et al., 2013) by TopHat (version TopHat v1.0.12; Trapnell et al., 2009), with parameters to avoid identification of splice junctions and to allow strand-specific mapping. Gene expression was determined by the Htseq package using the GeneBank *C. pneumoniae* CWL029 annotation file and discarding reads mapping all multiple positions of the bacterial genome. 1,445,880 reads were mapped to the *C. pneumoniae* genome under normoxia (1,375 reads/gene) and 7,575,167 reads under hypoxia (7,200 reads/gene). Data were normalized using the RPKM conversion and differential expression analysis was done using the Bioconductor package NOISeq version 2.6.0 (Tarazona et al., 2015). The NOISeq-sim function included in the package allows for differential expression estimates in absence of replication by simulating replicates considering that reads counts follow a multinominal distribution (Tarazona et al., 2011). To obtain the highest possible yield of bacterial mRNA which is present only in low abundance in total RNA we sequence one sample per condition. Afterwards several candidate genes were verified by quantitative RT-PCR.

### Quantitative RT-PCR

Total RNA was isolated using the NucleoSpin RNA II kit (Macherey-Nagel) and reverse-transcribed into cDNA (RevertAid First Strand cDNA Synthesis kit, Thermo Fischer Scientific). PCR amplification was performed by using the LightCycler Detection System (Bioline). Relative quantification of rpe (forward GCCACTTTGTTCCGAACCTT; reverse CCGCTTGAACCCCACATTTT), trxB (forward AGCATTGTCCGTTCCGTAGA; reverse AGCAGCAAATACTCCAGGGA), zwf (forward GGATCTCGCGGCAATTTCTT; reverse TTGAACCGTTCCTGGACCAT), and cydA (forward CCTTCTGGGGAGTGGTCTTC, reverse CAACTCCCCTAGCCGTTACA) mRNA expression was performed against endogenous control 16S gene (forward TCGCCTGGGAATAAGAGAGA; reverse AATGCTGACTTGGGGTTGAG) using the 2^−ΔΔCT^ method.

### Genome copy number

DNA was isolated by using the QIAamp DNA Mini Kit (Qiagen). Cells of one 6-well were lysed with 200 μl PBS. Isolation was performed according to the supplier's protocol. Genome copies (GC) were determined at specific time points during the developmental cycle of *C. pneumoniae*. Isolated DNA was analyzed with 16S primers listed above. PCR amplification was performed by using the LightCycler Detection System (Bioline). Evaluation of the data was done with the LightCycler Data Analysis program (Roche).

### RNA interference

Transfection was performed according to Invitrogen manufacturer's guideline. In brief, RNAi experiments were performed in 6-well culture dishes using 20 μM siRNA (ATP5G1 Stealth RNAi and Stealth RNAi negative control, low GC, Life Technologies GmbH) and Lipofectamine transfection reagent (Life Technologies GmbH) under normoxic conditions. HEp-2 cells were pre-treated for 24 h with siRNA prior to infection. Three different Stealth siRNAs for ATP5G1 (ATP5G1HSS141315, ATP5G1HSS141316, ATP5G1HSS182194) were used. Knockdown efficacy was analyzed by qRT-PCR at 48 hpi: ATP5G1HSS141315 (86%), ATP5G1HSS141316 (99%), ATP5G1HSS182194 (99%).

### Measurement of ROS

5-(and-6)-chloromethyl-2′,7′-dichlorodihydrofluorescein diacetate, acetyl ester (CM-H_2_DCFDA; Invitrogen GmbH) was used as an indicator for reactive ROS in cells. CM-H_2_DCFDA was reconstituted at 5 mM in dimethyl sulfoxide (DMSO) (Sigma-Aldrich) and then diluted with Hank's balanced salt solution (HBSS) (PAA Laboratories GmbH) to 5 μM. Cells were washed twice with HBSS, incubated with 5 μM CM-H_2_DCFDA for 45 min at 37°C, followed by further washing. Passive Lysis Buffer 5x (Promega) was diluted 1:5 with distilled water and protease inhibitor cocktail (Roche Diagnostics GmbH). CM-H_2_DCFDA-labeled cells were lysed in 400 μl lysis buffer. After centrifugation, 100 μl of the supernatant were added to 96-well tissue culture plate. Resulting fluorescence was monitored using a microplate reader (Infinite M200, Tecan) with excitation and emission wavelengths of 495 and 520 nm, respectively. Signal was quantified as relative fluorescence units (RFU).

### Mitochondrial membrane potential

Tetramethylrhodamine ethyl ester (TMRE) mitochondrial membrane potential assay kit (Abcam) was used for fluorescent labeling of mitochondria in living cells. Cells were incubated with 10 nM TMRE for 5 min, allowing for the positively charged dye to accumulate in negatively charged mitochondria. Analysis of mitochondrial membrane potential was performed under normoxic and hypoxic conditions. Mitochondrial fluorescence was detected in four emission channels by two-photon laser excitation and analyzed as described previously (Szaszak et al., 2011). The ionophore uncoupler of oxidative phosphorylation FCCP [carbonyl cyanide 4-(trifluoromethoxy) phenylhydrazone] was used beforehand to prove that the TMRE staining was specific. FCCP eliminated the mitochondrial membrane potential in a dose dependent manner as analyzed with a microplate reader (fluorescence intensity without FCCP = 30,520; 100 nM FCCP = 19,196, 1 μM FCCP = 18,018; 10 μM FCCP = 7,915).

### Two-photon laser scanning microscopy

For two-photon microscopy, 5 × 10^5^ HEp-2 cells were grown on 40 mm coverslips and infected with *C. pneumoniae* as described above. Coverslips were examined in a MiniCeM chamber for microscopy (JenLab). During microscopic measurements, cells were incubated in a custom-made micro-incubator with adjustable gas concentrations, allowing for measurements under normoxic and hypoxic conditions. NAD(P)H autofluorescence was imaged with a two-photon laser scanning microscope (DermaInspect, JenLab). Autofluorescence was excited at 730 nm with a tunable infrared titanium-sapphire femtosecond-laser (710–920 nm tuning range, MaiTai, Spectra Physics), with a Chroma 640DCSPXR dichroic mirror (AHF analysentechnik AG). A 40×/1.3 Plan-Apochromat oil-immersion objective (Zeiss) was used. Residual excitation light was blocked by a blue emission filter (BG39, Schott AG). FLIM data were collected with a time-correlated single-photon counting (TCSPC) system (PMH-100-0, SPC-830, Becker & Hickl) for 49.7 s per image. Fluorescence lifetimes were analyzed using the SPCImage software version: 3, 9, 4 (Becker & Hickl). The visual field was 110 × 110 μm^2^ corresponding to 256 × 256 pixels. For image analysis, a region of interest (ROI) was selected within the chlamydial inclusion, nucleus and mitochondria. Three cells of a visual field were analyzed from 10 visual fields per chamber of three independent measurements. Lifetime decay curves were fitted to a double exponential decay model. The instrument response function (IRF), which was included in the fit model, was measured from the second harmonic generation signal of beta-barium-borate crystal. For fluorescence intensity analysis of FLIM pictures, photon counts values of selected ROIs from three different cells were determined by SPCImage software.

### Measurement of OCR

To analyze extracellular flux, cells were seeded at a density of 1.5 × 10^4^ or 3 × 10^4^ cells/well, 48 h and 24 h prior to the assay in a XF24 Cell Culture Microplate (Seahorse Bioscience), respectively. The day before the assay, the XF assay cartridge was filled with 1 mL XF Calibrant Solution and incubated overnight without CO_2_ at 37°C. XF assays were performed in XF Assay Medium (4.5 g/l glucose; 100 mM Na-pyruvate; pH 7.4). Cells were washed twice with 600 μl assay medium overlayed with 475 μl assay medium, and finally equilibrated in a non-CO_2_ incubator for 1 h before measurement of oxygen consumption rates (OCRs). Reagent compounds of the XF Cell Mito Stress Kit were diluted according to the supplier's protocol. Ports A-C of the XF assay cartridge were filled with 75 μl of the respective compound prior to OCR analysis.

To exclude that cycloheximide which was used in *C. pneumoniae* infection depresses the host metabolism, we tested uninfected HEp-2 cells with or without the addition of cycloheximide (0.1 μg/mL) in a preliminary experiment. Seahorse analysis with or without cycloheximide did not show a difference in the OCR values (data not shown).

### Statistical analysis

Data are indicated as mean ± standard error of the mean (SEM). For statistical evaluation GraphPad Prism 6 was used. Statistical analysis was performed with a one tailed, unpaired Student *t-*test comparing two groups. When comparing three groups or more, one-way ANOVA with Sidak's correction for multiple comparisons was used. *p* ≤ 0.05 were considered as statistically significant.

### Data accession

RNA sequencing data was deposited in the Gene Expression Omnibus (GEO) under the accession number GSE94697.

## Results

### *C. pneumoniae* influences mitochondrial function of infected host cells

**Figure 1:**
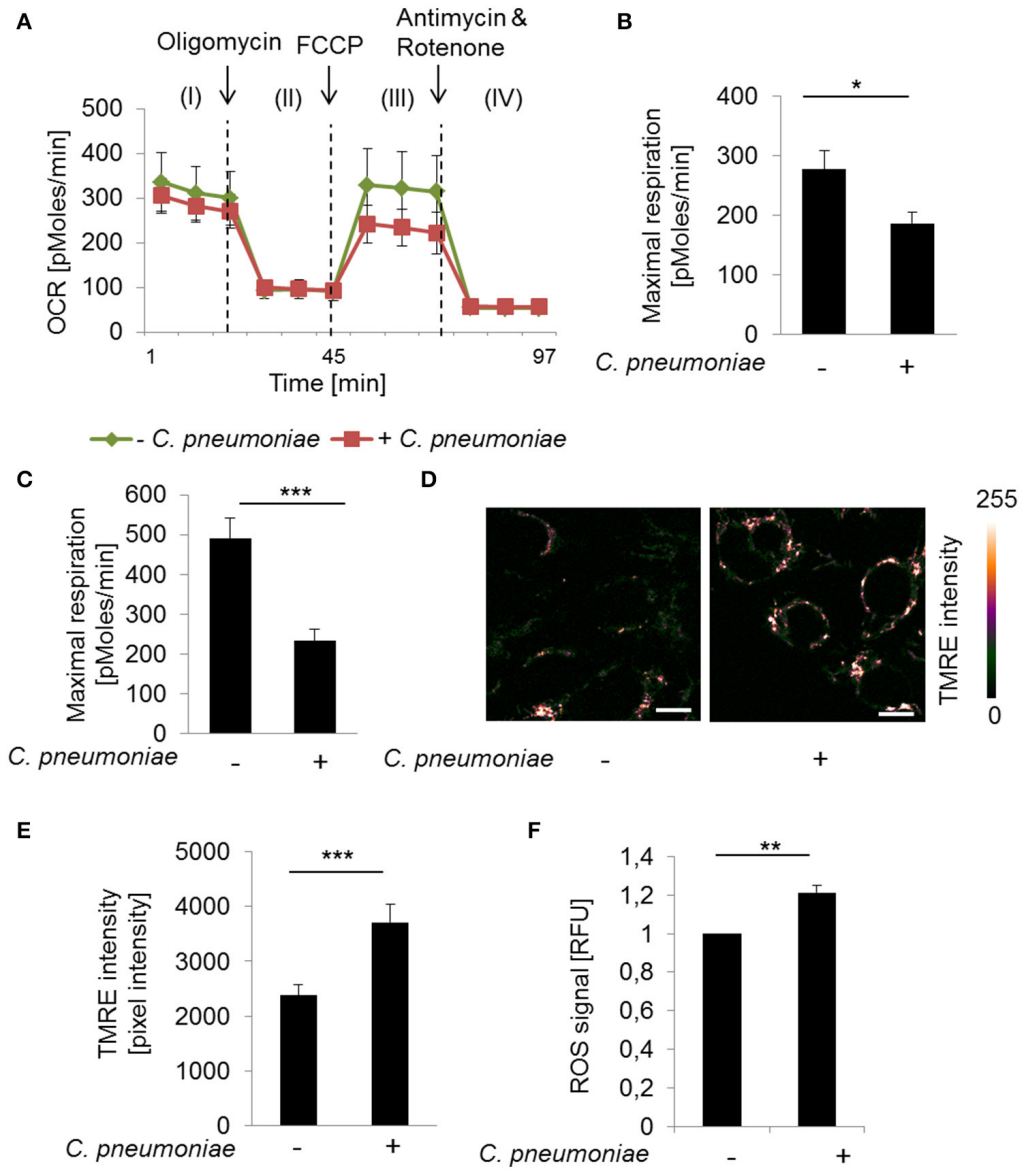
*C. pneumoniae* infection alters mitochondrial function of host cells. **(A)** HEp-2 cells were infected with *C. pneumoniae* under normoxia. The oxygen consumption rate (OCR) was measured by Mito Stress Test Kit at 24 hpi. I: Basal respiration, II: ATP production, III: Maximal respiration, IV: Non-mitochondrial oxygen consumption. **(B)** Maximal respiration derived from the OCR at 24 hpi (^*^p ≤ 0.05; *n* = 2) and **(C)** 48 hpi (^***^p ≤ 0.001; *n* = 3). **(D)** HEp-2 cells were infected with *C. pneumoniae* under normoxia. Tetramethylrhodamine ethyl ester (TMRE) was applied 48 hpi to analyze mitochondrial membrane potential. Color-coded images of fluorescence intensity analyzed by two-photon laser excitation (scale bar = 10 μm). **(E)** Quantitative analysis of TMRE intensity (^***^p ≤ 0.001; *n* = 3). **(F)** Reactive oxygen species (ROS) were measured in *C. pneumoniae*-infected HEp-2 cells under normoxia 24 hpi (^**^p ≤ 0.01; *n* = 3).

To characterize infection induced changes on mitochondrial respiration Seahorse XF Cell Mito Stress Test was performed. *C. pneumoniae* infection resulted in a significantly decreased maximal respiration compared to non-infected control cells at 24 and 48 hpi (Figures 1A–C), which is characteristic for mitochondrial dysfunction. Therefore, we performed TMRE- (tetramethylrhodamine ethyl ester) staining to visualize mitochondrial activities in *C. pneumoniae*-infected cells in more detail. Infected cells were analyzed by two-photon microscopy, allowing the analysis of the mitochondrial membrane potential in living cells under real-time conditions. A significant increase in the mitochondrial TMRE intensity, as a marker of mitochondrial hyperpolarization, was observed in *C. pneumoniae*-infected cells compared to non-infected cells at 48 hpi (Figures 1D,E). In addition, the increase in the mitochondrial membrane potential in *C. pneumoniae*-infected cells was associated with an increased ROS formation (Figure 1F). These changes point out that *C. pneumoniae* infection is accompanied by a mitochondrial dysfunction.

### Mitochondrial dysfunction affects chlamydial growth

**Figure 2:**
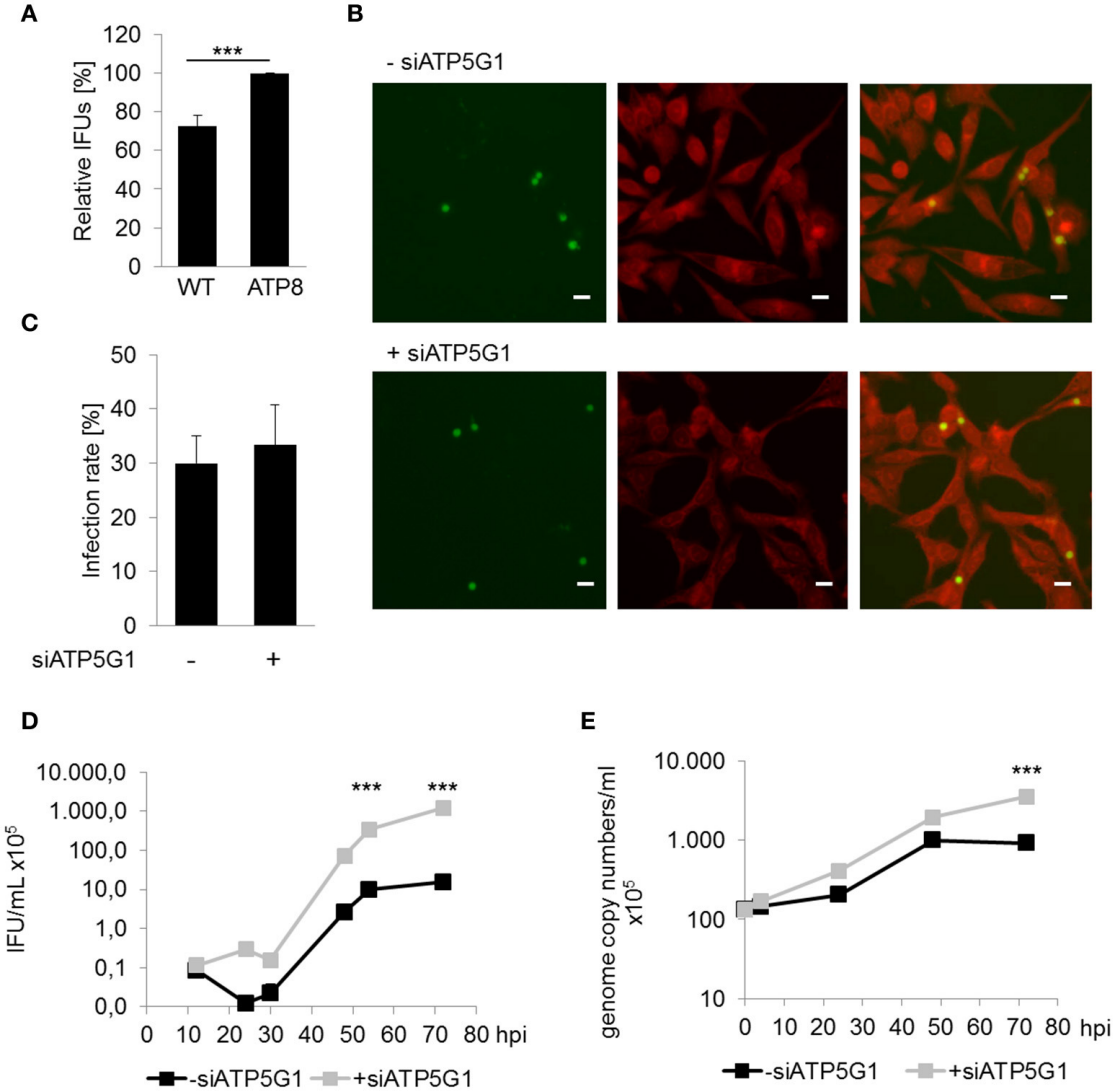
Mitochondrial dysfunction enhances chlamydial recovery rates. **(A)** Recoverable *C. pneumoniae* infection of mice fibroblasts wild type strain (WT) and carrying a mtDNA mutation (ATP8) (^***^p ≤ 0.001; *n* = 7). **(B)** *C. pneumoniae* infection in negative siRNA treated and ATP5G1 knockdown cells at 48 hpi. Direct immunofluorescence staining of chlamydial inclusions (green) and host cells (red) (scale bar = 10 μm). **(C)** *C. pneumoniae* infection rate of negative siRNA treated and ATP5G1 knockdown cells. **(D)** Recoverable *C. pneumoniae* from negative siRNA treated and ATP5G1 knockdown cells (^***^p ≤ 0.001 compared to negative control; *n* = 3). **(E)** Genome copy numbers of *C. pneumoniae* from negative siRNA treated and ATP5G1 knockdown cells (^***^p ≤ 0.001 compared to negative control; *n* = 3).

To assess the relevance of mitochondrial dysfunction for *C. pneumoniae* growth, fibroblasts from conplastic mice strains harboring mtDNA mutations were infected with *C. pneumoniae* to determine if altered mitochondrial performance (Yu et al., 2009), has an impact on chlamydial growth. Cells carrying a mutation in the F_0_-subunit of the ATP synthase (ATP8) showed a small, but significant increase, in the infectious progeny of *C. pneumoniae* (33.8 ± 12.6 × 10^5^ IFU/mL) compared to wild type (WT) cells (23.6 ± 8.56 × 10^5^; Figure 2A).

In addition, HEp-2 cells were pretreated with small interfering RNA (siRNA) targeting the F_0_-subunit of the host cell ATP synthase. Quantification of the infection rates revealed no significant changes due to the siRNA-dependent inhibition of ATP5G1 (Figures 2B,C). Furthermore, inclusion size was comparable between ATP5G1 siRNA (5.84 ± 0.14 μm) and siRNA negative control (5.83 ± 0.15 μm) at 48 hpi. However, recovery rates and genome copy numbers of *C. pneumoniae* increased significantly in cells treated with ATP5G1 siRNA compared to the siRNA negative controls over 72 hpi (Figures 2D,E and Figure S1). By calculating the generation time during the exponential log phase (24–48 hpi), we found that the doubling rate was comparable between both conditions (10.7 h in ATP5G1 siRNA vs. 10.4 h in siRNA negative control). Taken together, these experiments show that *C. pneumoniae* growth is supported in cells with mitochondrial dysfunction.

### Low oxygen as a physiological model to study mitochondrial dysfunction

**Figure 3:**
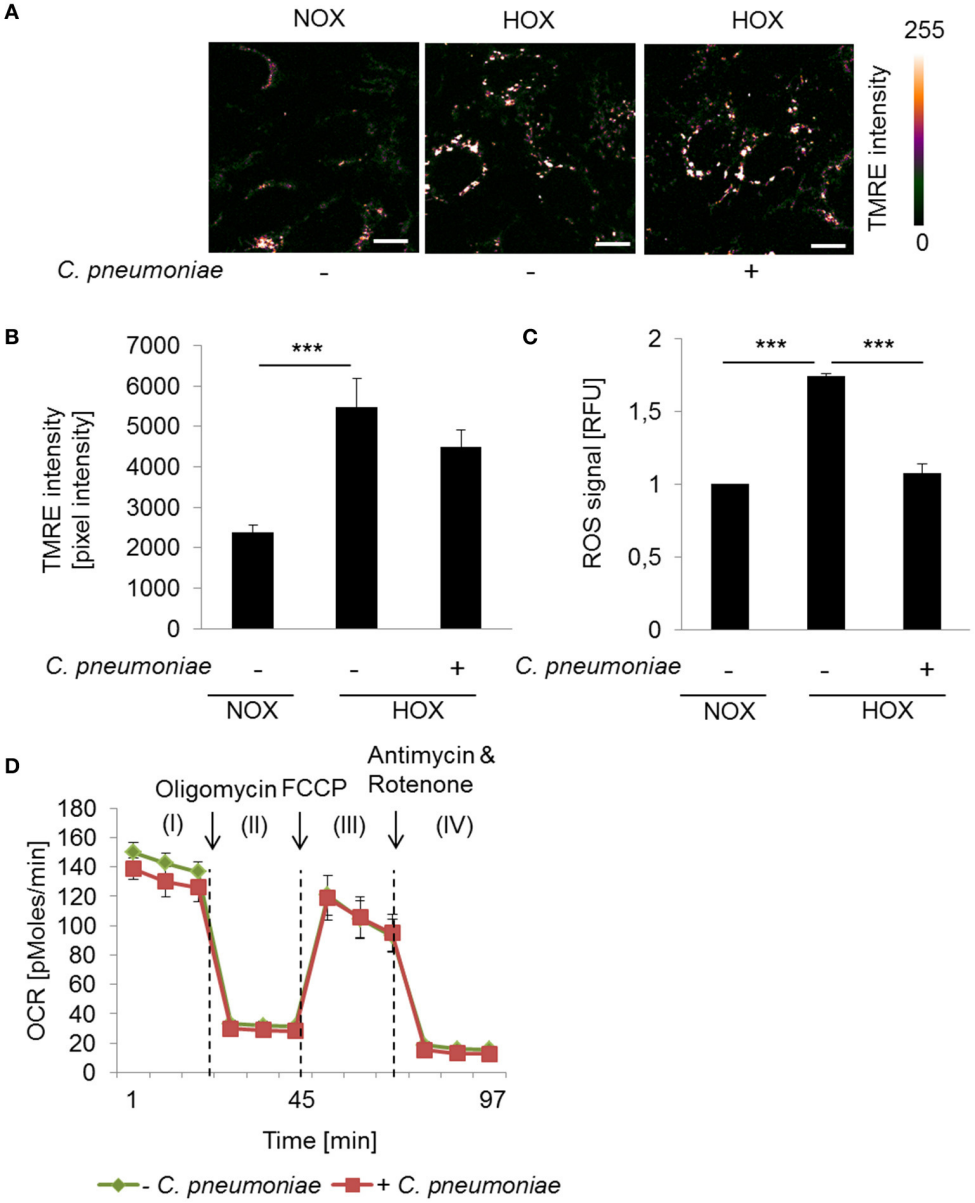
Mitochondrial function in *C. pneumoniae* infection under hypoxia. **(A)** HEp-2 cells were infected with *C. pneumoniae* under hypoxia and compared to normoxic control. Tetramethylrhodamine ethyl ester (TMRE) was applied 48 hpi. Color-coded images of the fluorescence intensity was analyzed by two-photon laser excitation (scale bar = 10 μm). **(B)** Quantitative analysis of TMRE intensity (^***^p ≤ 0.001; *n* = 3). **(C)** Reactive oxygen species (ROS) were measured in *C. pneumoniae*-infected HEp-2 cells under hypoxia 24 hpi, compared to normoxic control (^***^p ≤ 0.001; *n* = 3). **(D)** HEp-2 cells were infected with *C. pneumoniae* under hypoxia. The oxygen consumption rate (OCR) was measured by Mito Stress Test Kit at 24 hpi. I: Basal respiration, II: ATP production, III: Maximal respiration, IV: Non-mitochondrial oxygen consumption.

To further analyze the impact of the mitochondrial activity on intracellular growth and progeny of *C. pneumoniae* in a more physiological setting, we analyzed the cells in a low oxygen environment (hypoxia, 2% O_2_). As expected, enhanced TMRE intensity and ROS formation was detected under hypoxic compared to normoxic conditions in non-infected cells (Figures 3A–C), reflecting the hypoxia-induced mitochondrial dysfunction that is known from hypoxic tumor cells. Hence, in *C. pneumoniae*-infected cells under hypoxic conditions mitochondrial membrane potential did not increase additionally (Figures 3A,B). As a consequence, hypoxic non-infected and infected cells display no differences in maximal respiration (Figure 3D). ROS formation in *C. pneumoniae*-infected cells under hypoxic conditions was significantly reduced (Figure 3C).

### Hypoxia enhances metabolic activity of *C. pneumoniae* and host cell

To understand how *C. pneumoniae* growth is favored by hypoxia-induced mitochondrial dysfunction, we applied a non-targeted metabolic approach applying ultra-high-resolution mass spectrometry (ICR/FT-MS) and ultra-performance liquid chromatography mass spectrometry (RP UPLC-MS and HILIC-MS) of total cell extracts to characterize the metabolic needs of *C. pneumoniae*. In unsupervised PCA models and PLS-DA models we showed separation of non-infected and *C. pneumoniae*-infected samples both in normoxia and hypoxia, demonstrating the abundance of different metabolic profiles (Figure S2). The discriminative annotated metabolites of the PLS-DA model between normoxia and hypoxia were mapped into the KEGG pathways and categorized according to the main metabolic classes (Table S1). Comparing normoxia to hypoxia the highest metabolic variations were detected in the carbohydrate metabolism of non-infected cells. The analysis revealed a decrease in the carbohydrate variations in infected cells between normoxia and hypoxia. On the one hand, this might indicate that the metabolism in *C. pneumoniae*-infected cells under normoxia and hypoxia are comparable. On the other hand, the high variations in non-infected cells might simply not be seen in infected cells because *C. pneumoniae* uses the metabolites for its own metabolism and growth.

To prove these assumptions, host and chlamydial metabolic changes were separately analyzed during hypoxia-induced mitochondrial dysfunction using fluorescence imaging by two-photon microscopy of the autofluorescent metabolic coenzyme NAD(P)H.

**Figure 4:**
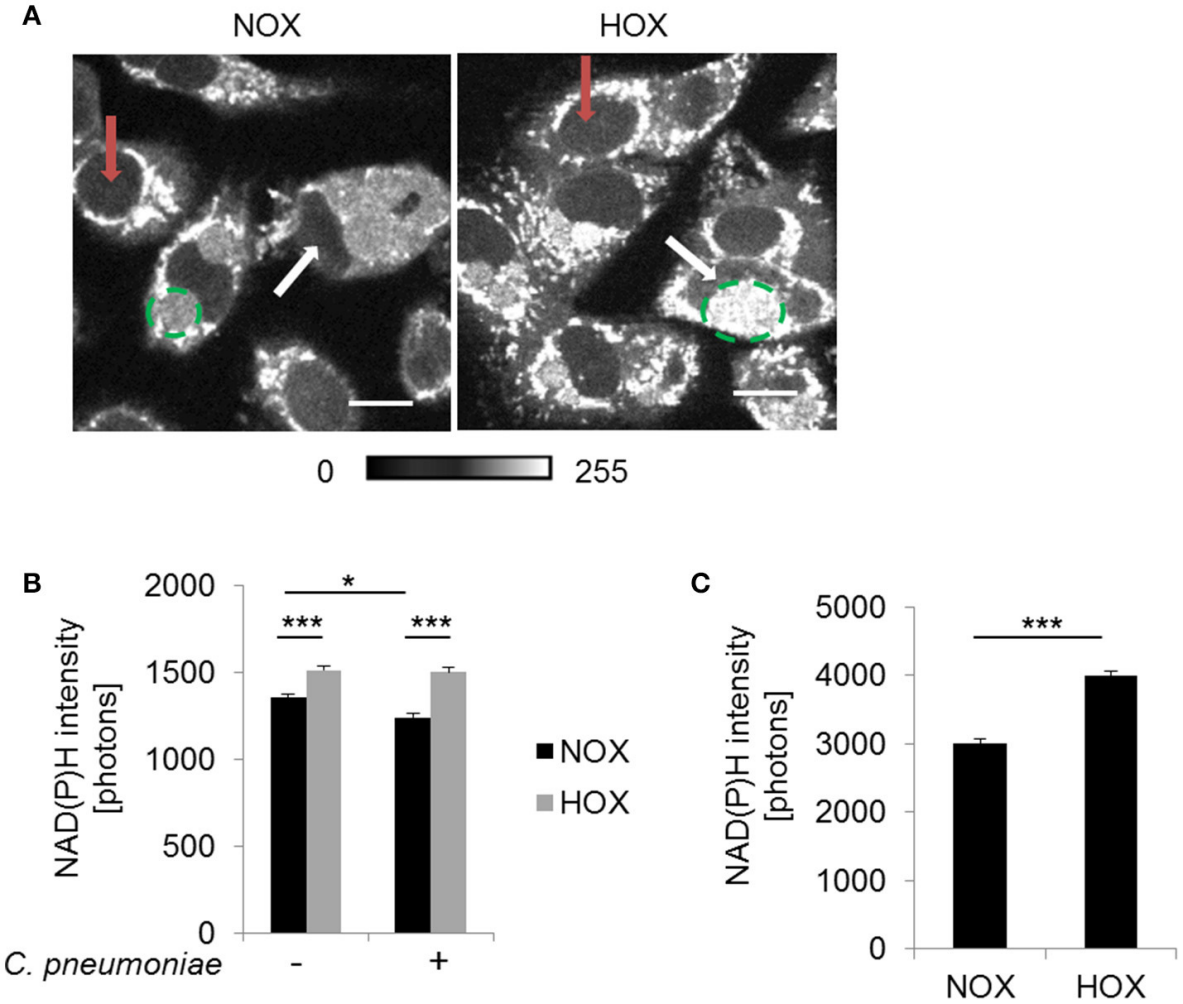
NAD(P)H fluorescence intensity under normoxia and hypoxia. HEp-2 cells were infected with *C. pneumoniae* for 48 h. **(A)** Gray-scale images of NAD(P)H fluorescence intensity (scale bar = 10 μm). Quantitative analysis of NAD(P)H intensity in the nucleus **(B)** and the chlamydial inclusion **(C)** (^*^p ≤ 0.05; ^***^p ≤ 0.001; *n* = 3). Red arrows show nuclei of non-infected cells and white arrows nuclei of infected cells. Chlamydial inclusions are marked by dashed green lines.

Metabolic changes of the host cytosol are represented by the nucleus, since NAD(P)H is freely diffusible through the nuclear pore. NAD(P)H autofluorescence intensity was analyzed in the nucleus of *C. pneumoniae*-infected cells at 48 hpi under normoxia and hypoxia (Figure 4A). NAD(P)H intensity was significantly enhanced in *C. pneumoniae*-infected cells under hypoxia compared to the respective normoxic control (Figures 4A,B).

Under hypoxia, chlamydial inclusions showed increased NAD(P)H autofluorescence intensity at 48 hpi (Figure 4A). Quantitative analysis revealed a significantly higher NAD(P)H intensity under hypoxia compared to normoxia (Figure 4C). NAD(P)H from the host is acquired to overcome the lack of biosynthetic pathways for this metabolic coenzymes, indicating an increased metabolic activity of *C. pneumoniae* under hypoxia.

### Transcriptional regulation of *C. pneumoniae* under hypoxia

To further define the enhanced metabolic activity of *C. pneumoniae* under hypoxia-induced mitochondrial dysfunction a transcriptome screen was performed. Analysis of differentially expressed chlamydial candidate genes by NOISeq-sim revealed 153 upregulated and 18 downregulated genes 24 hpi (Table S2). Regarding the lack of replications in the transcriptome screen we applied a bioinformatic method which takes into account both the values of fold-change and the difference between experimental groups. According to these values and comparing them with a noise distribution, the probability of differential expression is computed for each gene. In addition, we validated selected genes using qRT-PCR to receive a reliable picture of the chlamydial transcriptome (Figure S3). Most of the upregulated genes under hypoxia belong to the transcriptional and translational machinery or have unknown function. Moreover, transporters show increased expression under hypoxia such as the ATP/ADP translocase (Cpn0614) responsible for NAD uptake. Within the group of metabolic genes, numerous genes belonging to the energy metabolism and nucleotide metabolism were upregulated under hypoxia (Table 1, Table S2, Figure S3) indicating enhanced chlamydial metabolic activity under hypoxia.

**Table 1:** Candidate genes of the chlamydial energy metabolism and transporters which are upregulated under hypoxia compared to normoxia in transcriptome analysis.

**Gene**	**Name**	**Product**	**Read count RPKM**	
			**Normoxia**	**Hypoxia**
**ENERGY METABOLISM**				
Cpn0088	atpA	V-type ATP synthase subunit A	469	785
Cpn0102	cydA	cytochrome D ubiquinol oxidase subunit I	1,178	2,530
Cpn0106	phoH	ATPase	1,056	1,515
Cpn0185	rpe	ribulose-phosphate 3-epimerase	134	283
Cpn0238	zwf	glucose-6-phosphate 1-dehydrogenase	1,134	1,977
Cpn0624	gapA	glyceraldehyde-3-phosphate dehydrogenase	2,291	3,241
**TRANSPORTER**				
Cpn0023	yjjK	ABC transporter ATPase	872	1,532
Cpn0061	ptsN_2	PTS IIA protein %2B HTH DNA-binding domain	410	733
Cpn0231	tauB	nitrate%2Firon ABC transporter ATPase	934	1,470
Cpn0290		Na-dependent transporter	215	395
Cpn0486		proline permease	208	665
Cpn0536	dagA_1	D-Ala%2FGly permease	717	1,143
Cpn0604	fliY	amino acid ABC transporter substrate-binding protein	2,852	4,541
Cpn0614	adt_2	ADP/ATP translocase	1,418	2,137

*Gene coverage was normalized using RPKM conversion. Ranking of differentially expressed candidate genes were identified by NOIseq (n = 1)*.

Our data show that mitochondrial dysfunction, characterized by mitochondrial hyperpolarization and elevated ROS levels, turned out to be a regulator of host metabolism affecting *C. pneumoniae* growth and progeny. The hypoxic phenotype causes enhanced activity of the chlamydial metabolism indicated by transcriptome data and NAD(P)H autofluorescence intensity under conditions of mitochondrial dysfunction, which promote enhanced chlamydial growth.

## Discussion

Abnormalities in the function of mitochondria are defining a mitochondrial dysfunction, such as impairment of the respiratory chain or mitochondrial hyperpolarization leading to increased mitochondrial membrane potential, preventing apoptosis (Michelakis, 2008; Brand and Nicholls, 2011). Further, mitochondrial dysfunction induces ROS (Murphy, 2009). ROS production during *C. trachomatis* infection is beneficial for bacterial development through activation of caspase-1 and hypoxia-inducible factor-1α (HIF-1α) (Prusty et al., 2012). Therefore, we hypothesize that these features would be beneficial for chlamydial growth. It has been previously shown that *C. pneumoniae* infection of mouse macrophages induced mitochondrial dysfunction (Shimada et al., 2011). Here we show that *C. pneumoniae* infected HEp-2 cells exhibit a mitochondrial dysfunction, characterized by decreased maximal respiration, hyperpolarization of the mitochondrial membrane potential and enhanced ROS generation. The decrease in maximal respiration was specific for active chlamydial growth since heat-inactivated *C. pneumoniae* did not affect the maximal respiration (data not shown).

In previous studies it was shown that a mutation in the ATP synthase 8 (ATP8) alters the mitochondrial performance and increases ROS (Yu et al., 2009). Further, Schröder et al. defined that ATP8 mutation leads to a mitochondrial dysfunction, characterized by increased ROS generation and low ATP (Schroder et al., 2016). For additional characterization of ATP8 mutant cells, we tried to establish Seahorse XF Cell Mito Stress Test. It was difficult to maintain a homogenous cell monolayer, which is necessary to perform this technique. Therefore, the respiration rate was too low for validation. We found that *C. pneumoniae* growth is indeed promoted by a predominant mitochondrial dysfunction in fibroblasts carrying ATP8 mutation. Likewise, knockdown of ATP5G1 of the ATP synthase led to an increased infectious progeny and genome copy numbers. As the generation time during exponential log phase was comparable between ATP5G1 siRNA treated and control cells we assume that primarily a shorter lag phase accounts for the increase in infectious progeny. A shortened lag phase was also described by Juul et al. during *C. pneumoniae* growth under hypoxic conditions accounting for enhanced infectious progeny (Juul et al., 2007).

Inhibition of host ATP synthase increases the mitochondrial membrane potential (Rego et al., 2001) and switches ATP production to glycolysis (Brand and Nicholls, 2011). Recently, Chowdhury et al. reported that the increased energy demand of *C. trachomatis*-infected cells is provided by elongated mitochondria as a result of higher fusion/fission ratio and that mitochondrial ATP is essential for *C. trachomatis* growth (Chowdhury et al., 2017). In contrast, our data indicate that in *C. pneumoniae* infection mitochondrial dysfunction seems to be the preferred phenotypic state. Different metabolic characteristics might occur due to the different tissue tropism and differences in their genome, which requires an individual adaption of the two species. So far, no data is available to demonstrate if a mitochondrial dysfunction is involved in the growth of *C. pneumoniae* under *in vivo* conditions.

We used hypoxia as physiological model since tissue displays different oxygen concentrations varying from 14.5% in the alveolar space to 5.6% in the peribronchial tissue (Ryan and Hickam, 1952; Herold et al., 1998). Moreover, oxygen concentration further declines due to growth of intracellular pathogens and inflammatory processes (Kempf et al., 2005; Eltzschig and Carmeliet, 2011; Campbell et al., 2014). In previous studies it was demonstrated that low oxygen conditions are beneficial for the intracellular growth of *C. pneumoniae* (Juul et al., 2007; Rupp et al., 2007; Szaszak et al., 2013). Mitochondrial dysfunction could be of relevance for enhanced intracellular growth under hypoxia since an inadequate oxygen supply leads to a defective OXPHOS. ETC activity is altered under hypoxia due to the oxygen-dependent regulation of a cytochrome c oxidase (COX) subunit (Semenza, 2007). Low oxygen inhibits COX (Chandel et al., 1998), resulting in a premature electron transfer at complex III (Semenza, 2007), thereby increasing the amount of ROS under hypoxia (Bell et al., 2007). Further, hypoxia impairs the activity of the ATP synthase, leading to a hyperpolarization of mitochondrial membrane potential, thus causing a switch to glycolysis (Chandel et al., 1997; Gao and Wolin, 2008). The here induced changes in mitochondrial hyperpolarization and ROS by hypoxia might play a crucial role for chlamydial growth, since they promote glycolysis and increase cellular longevity.

We could show that a metabolic switch associated with a mitochondrial dysfunction occurs during *C. pneumoniae* growth under hypoxia in HEp-2 cells. Oxygen is needed to produce energy and plays a fundamental role in cell metabolism (Carreau et al., 2011). Thus, our metabolic screen revealed metabolic alterations between normoxic and hypoxic conditions. We observed in non-infected cells that of all metabolites the carbohydrate metabolism was influenced to the greatest extent between normoxic and hypoxic conditions. Ojcius et al. showed that an enhanced carbohydrate metabolism was connected with *C. psittaci* infection, compensating the increased energy burden in infected cells (Ojcius et al., 1998). Therefore, the high variations in the carbohydrate metabolism seem to be beneficial for *C. pneumoniae* growth since *C. pneumoniae* takes up and utilizes these metabolites. Hence, the metabolic variations identified in non-infected cells between normoxia and hypoxia were abrogated when cells are infected with *C. pneumoniae*. Evidence for enhanced uptake of metabolites by *C. pneumoniae* is provided by transcriptomic data, where chlamydial transporters show enhanced expression under hypoxia. Since *Chlamydia* lack biosynthetic pathways for NAD and NAD(P), *C. pneumoniae* depends on host cell derived NAD uptake, maintained by the ATP/ADP translocase (Fisher et al., 2013). Indeed, we could show by transcriptome analysis that the ATP/ADP translocase (Cpn0614) was upregulated under hypoxic conditions. So far it is not possible to prove the involvement of the transporters in the enhanced growth since suitable techniques for genetic modification in this organism are missing. Thus, we suggest that the increased level of NAD(P)H in the chlamydial inclusion indicated an increased need for this metabolic coenzyme leading to enhanced metabolic activity of *C. pneumoniae* under hypoxic conditions. In addition, this was accompanied with enhanced transcription of chlamydial genes related to glycolysis and PPP. With the applied bioinformatic method used to get the probability of differentially expressed genes from transcriptome screen without replication and the validation of selected genes in multiple replicates by qRT-PCR we received a reliable picture of the chlamydial transcriptome. The relevance of chlamydial metabolic capabilities was indicated by Engström et al. where inhibition of chlamydial glucose-6-phosphate metabolism reduced *C. trachomatis* progeny (Engstrom et al., 2015).

We observed an increase of NAD(P)H intensity in the nucleus in non-infected as well as *C. pneumoniae*-infected cells under hypoxia-induced mitochondrial hyperpolarization. Enhanced NAD(P)H autofluorescence indicates high glycolytic activity since the autofluorescence increases in a glucose-dependent manner (Evans et al., 2003). These results suggest that the enhanced host cell glycolysis, caused by the hyperpolarization induced switch from OXPHOS to glycolysis, promotes the increased growth of *C. pneumoniae* under hypoxic conditions.

This study addresses for the first time that an increased growth of *C. pneumoniae* arises in cells with impaired mitochondrial function. We suggest that enhanced growth of *C. pneumoniae* under hypoxia is the result of hypoxia-induced mitochondrial dysfunction and the associated metabolic switch. However, we cannot exclude that the decrease in ROS under hypoxia during *C. pneumoniae* growth is responsible for the enhanced growth. Since the effects of our observations are moderate further characterization is still required to prove the biological relevance of our findings in upcoming studies using *in vivo* experiments.

## Author contributions

NK, IK, CM, MS, KS, JR, PS-K: designed experiments; NK, IK, CM, MS, KS, JR: interpreted data; NK, IK, CM: performed experiments; CM, PS-K: performed and analyzed MS experiments; TW, RL, AC, TR: established and performed bioinformatic analysis of transcriptomic data; NK, IK, JR: wrote the manuscript. All authors contributed in preparing the final version.

### Conflict of interest statement

The authors declare that the research was conducted in the absence of any commercial or financial relationships that could be construed as a potential conflict of interest.
