# Supplementary material for: Growth of Chlamydia pneumoniae Is Enhanced in Cells with Impaired Mitochondrial Function
Source: Front Cell Infect Microbiol. 2017 Dec 5;7:499. doi: 10.3389/fcimb.2017.00499 (PMC5723314; doi:10.3389/fcimb.2017.00499)
Supplement: Supplementary file 1 [file Image1.pdf]

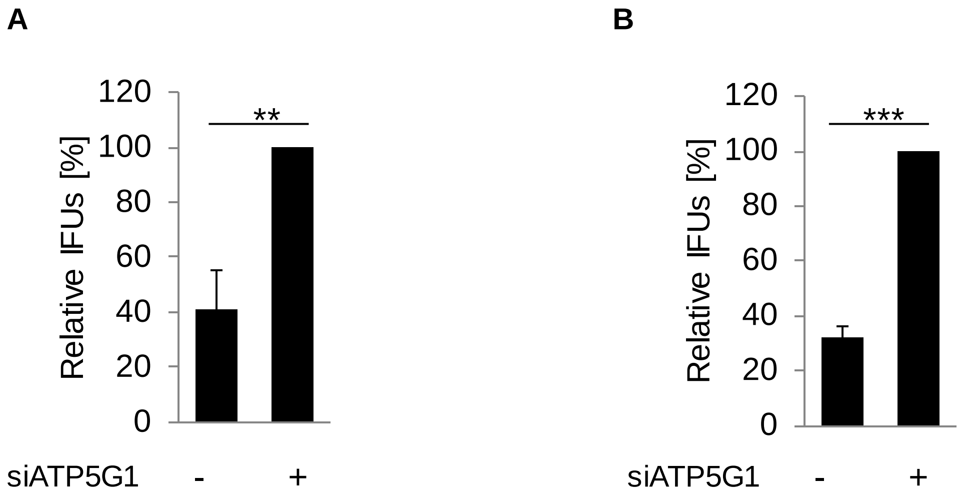

Supplementary Figure 1: Effect of second and third siRNA on chlamydial recovery.

Recoverable *C. pneumoniae* from negative siRNA treated and ATP5G1 knockdown cells (A) ATP5G1HSS141316 and (B) ATP5G1HSS182194 (**$p \leq 0.01$; ***$p \leq 0.001$; n=3).
