# Supplementary material for: Growth of Chlamydia pneumoniae Is Enhanced in Cells with Impaired Mitochondrial Function
Source: Front Cell Infect Microbiol. 2017 Dec 5;7:499. doi: 10.3389/fcimb.2017.00499 (PMC5723314; doi:10.3389/fcimb.2017.00499)
Supplement: Supplementary file 2 [file Image2.pdf]

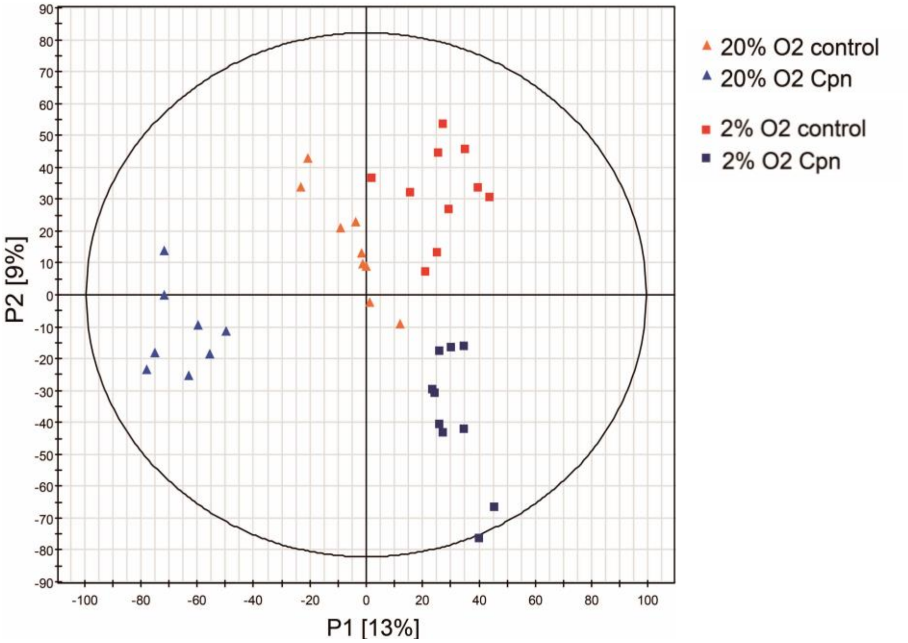

Supplementary Figure 2: Non-targeted metabolic screen of non-infected and *C. pneumoniae*-infected HEp-2 cells under normoxia and hypoxia.

Score plots of PLS-DA model of ICR/FT-MS data of non-infected and *C. pneumoniae*-infected HEp-2 cells under normoxia and hypoxia. The conditions could be differentiated based on the expression of their metabolites patterns (n=10).
