# Supplementary material for: Growth of Chlamydia pneumoniae Is Enhanced in Cells with Impaired Mitochondrial Function
Source: Front Cell Infect Microbiol. 2017 Dec 5;7:499. doi: 10.3389/fcimb.2017.00499 (PMC5723314; doi:10.3389/fcimb.2017.00499)
Supplement: Supplementary file 3 [file Image3.pdf]

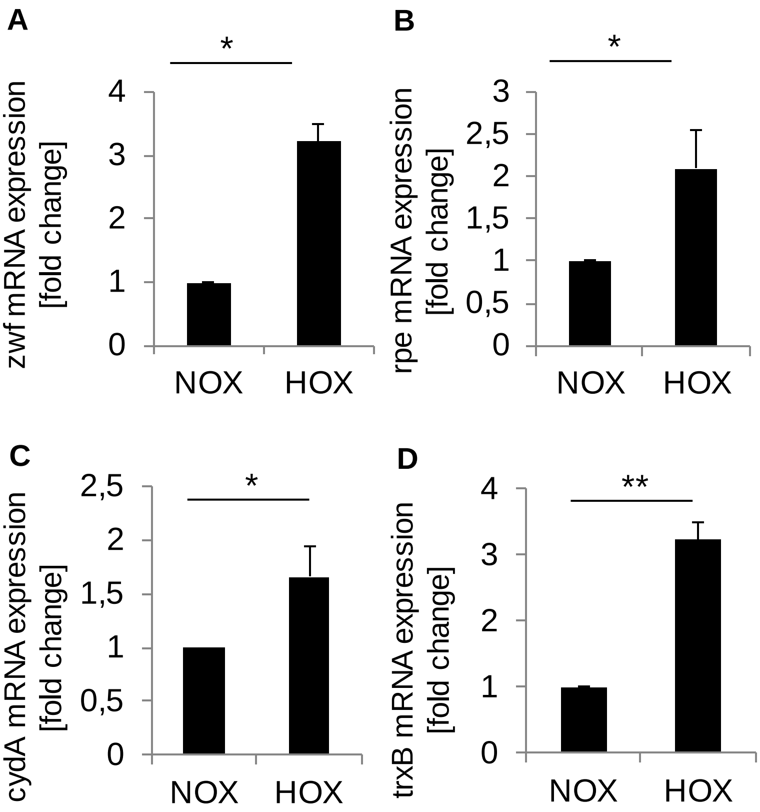

Supplementary Figure 3: Validation of selected chlamydial genes upregulated in the transcriptome screen.

(A) Glucose-6-phosphate 1-dehydrogenase (zwf) (n=4), (B) ribulose-phosphate 3-epimerase (rpe) (n=5), (C) cytochrome D ubiqinol oxidase subunit I (cydA) and (n=8) (D) thioredoxin reductase (trxB) (n=4) of *C. pneumoniae* grown in HEp-2 cells under normoxia or hypoxia 24 hpi were analyzed by qRT-PCR and normalized to 16S rRNA (*$p \leq 0.05$; **$p \leq 0.01$).
